# Supplementary material for: The genetic basis of energy conservation in the sulfate-reducing bacterium Desulfovibrio alaskensis G20
Source: Front Microbiol. 2014 Oct 31;5:577. doi: 10.3389/fmicb.2014.00577 (PMC4215793; doi:10.3389/fmicb.2014.00577)
Supplement: Supplementary file 1 [file Presentation1.PDF]

Proposed Scenarios for Electron Flow During Sulfate Reduction By *Desulfovibrio alaskensis* G20

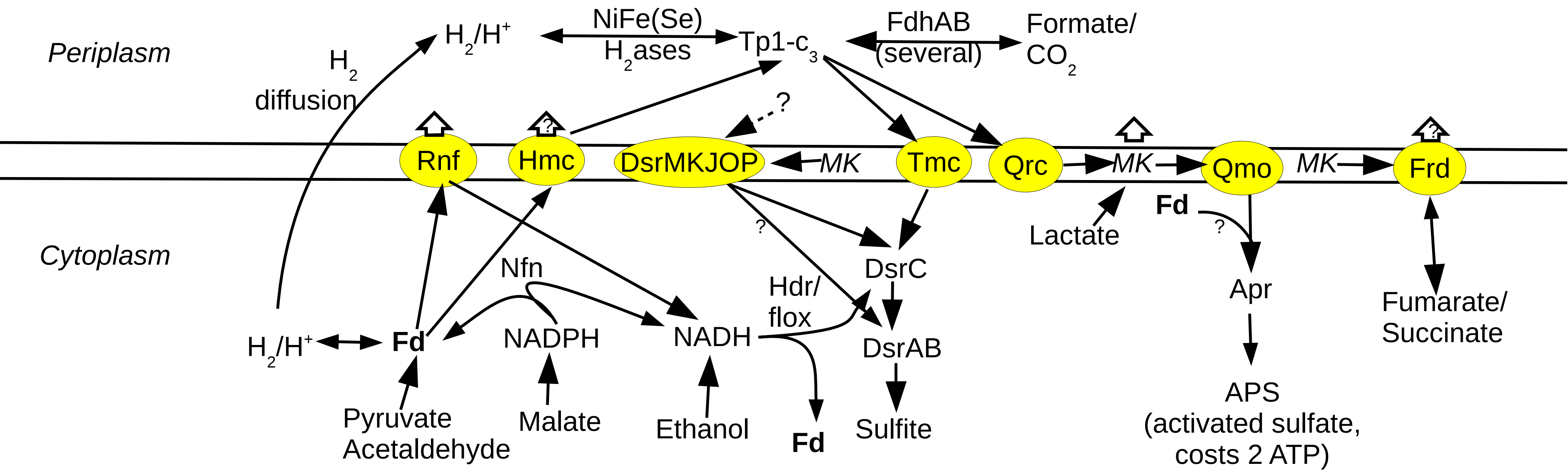

For these scenarios, we make *simplifying and uncertain* assumptions about Qmo and DsrMKJOP:

2 electrons to reduce APS to sulfite:

- 1 from Fd
- 1 from MK

6 electrons to reduce sulfite to sulfide:

- 2 from DsrC (which can be from MK via DsrMKJOP)
- 4 from MK via DsrMKJOP

Overall, to reduce 1 molecule of APS, need reduced forms of

- 0.5 Fd + 1 DsrC + 2.5 MK

A recurring issue with this model is: where does all the reduced MK for sulfite reduction come from?

- Alternatively, can all 6 electrons for sulfite reduction come from DsrC, perhaps by reversibility of DsrMKJOP?

Scenario for Pyruvate/Sulfate

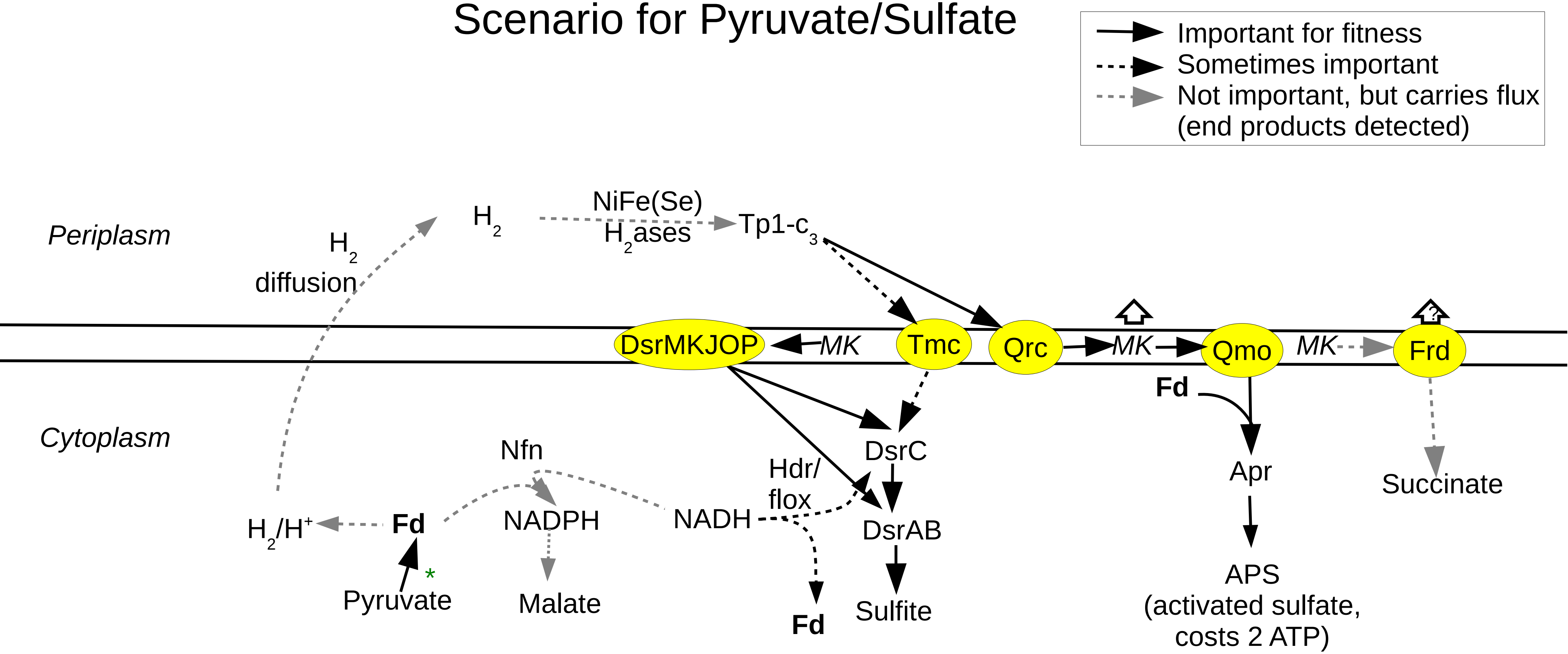

Per sulfate reduced:

4 pyruvate → 4 acetyl-CoA + 4 reduced Fd

2 net ATP from substrate-level phosphorylation

Pyruvate can also be reduced to malate, converted to fumarate, and further reduced to succinate

This requires NADPH, which might be formed by Nfn

To reduce 1 APS, need: 2.5 reduced MK, 1 reduced DsrC, 0.5 reduced Fd

So need to convert 3.5 Fd → 1 DsrC + 2.5 MK

DsrC is reduced by Hdr/flox (which requires NADH), Tmc (which requires reduced Tp1-c_3), or DsrMKJOP (from reduced MK)

NADH is reduced by Rnf (not shown) or by Hdr/flox (in reverse)?

Tp1-c_3 is reduced by hydrogen cycling, formate cycling (not shown), or possibly Hmc (not shown)

MK is reduced by Qrc, which requires reduced Tp1-c_3

Scenario for Lactate/Sulfate

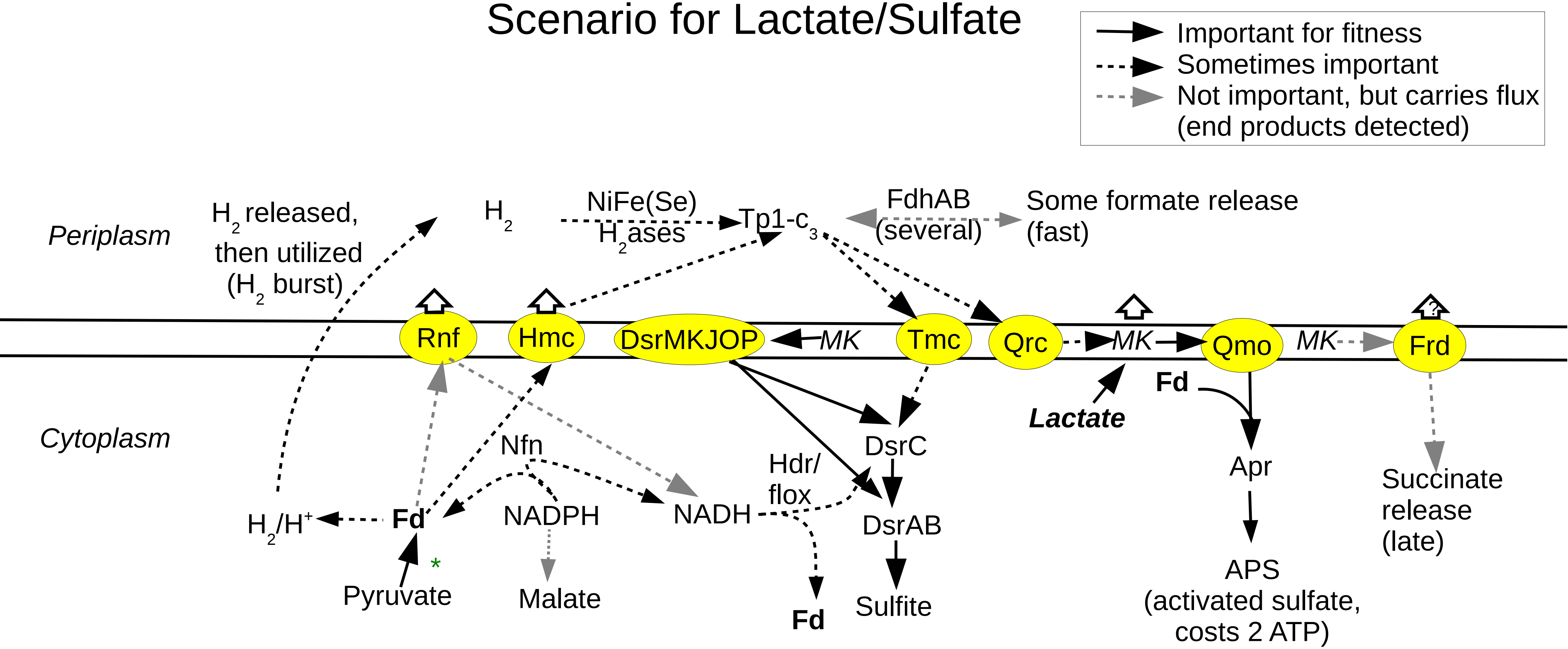

Per sulfate reduced:

- 2 lactate → 2 pyruvate + 2 reduced MK
- 2 pyruvate → 2 acetyl-CoA + 2 reduced Fd
- 0 net ATP from substrate-level phosphorylation

To reduce 1 APS, need: 2.5 reduced MK, 1 reduced DsrC, 0.5 reduced Fd

- So need to convert 1.5 Fd → 1 DsrC + 0.5 MK

0.5 MK from Tp1c_3 and Qrc, with Tp1c_3 reduced by Fd via H_2 cycling or Hmc

- Or, if >2 lactate oxidized per sulfate reduced, and Fd is converted to H_2, then excess MK is achieved, and net ATP is increased because not all is used for activating APS

1 DsrC is reduced by Tmc (from reduced Tp1c_3) or DsrMKJOP (via Qrc and MK)

Some pyruvate is reduced to malate, which is converted to fumarate, and reduced to succinate

- The source of NADPH is unclear (Nfn & Rnf?), as is the source of excess MK

Energy conservation by Qmo and by H_2 release, H_2 cycling, Hmc, or Rnf with Hdr/flox (lots of redundancy)

Scenario for Fumarate/Sulfate

Important for fitness
Sometimes important
Not important, but carries flux (end products detected)

Overview:

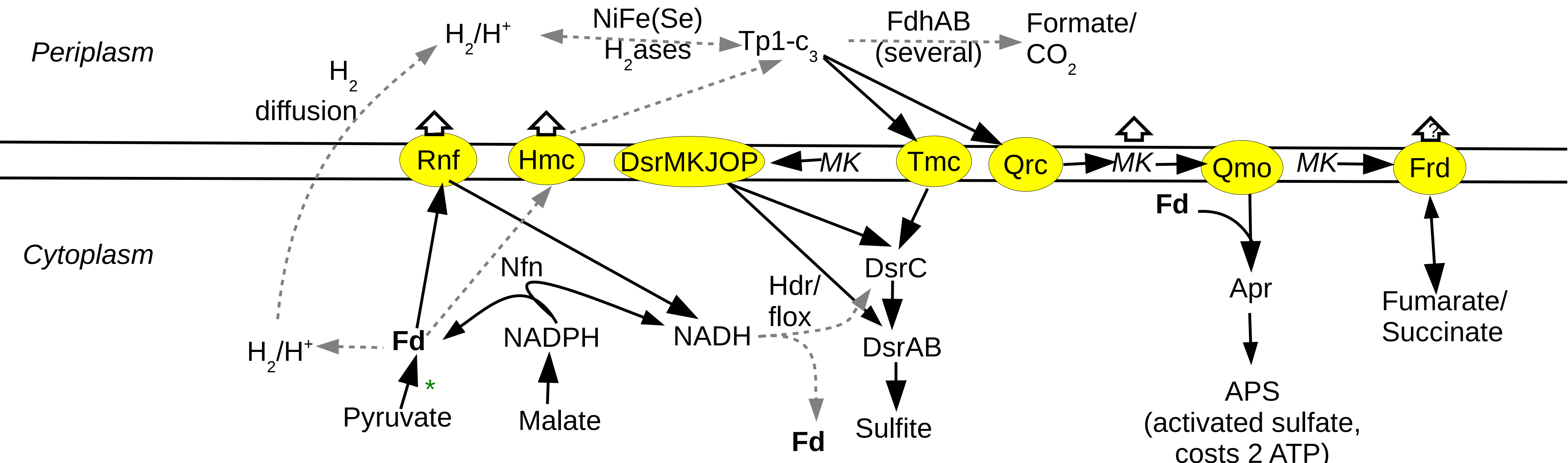

Per sulfate reduced:

- 2 fumarate ↔ 2 malate → 2 pyruvate + 2 reduced NADPH
- 2 pyruvate → 2 acetyl-CoA + 2 reduced Fd
- +0 net ATP from substrate-level phosphorylation

Fumarate can also be reduced to succinate, but we found no net succinate reduction:

- succinate appeared early and then disappeared. Nevertheless, Frd was important for fitness.

To reduce 1 APS, need 2.5 reduced MK, 1 reduced DsrC, 0.5 reduced Fd

Nfn converts NADPH to reduced NADH + reduced Fd

Neither Hdr/flox cluster was important for fitness, but it is expected to consume the NADH from Nfn (and Rnf)

- And it would produce DsrC. (Are hdr/flox-1,-2 redundant? Is there another path from NADH?)

The path to Tp1-c_3 is unclear but could involve hydrogen cycling or possibly Hmc (mean fitness = -0.1)

- (We did not measure H_2 release but assume that it occurs.)

Given reduced Tp1-c_3, Tmc could reduce DsrC, and Qrc could reduce MK

Energy conservation by Nfn, Rnf, Qrc/Qmo, and possibly hydrogen cycling or Hmc

Note: electron flow on malate/sulfate is probably very similar to this.

Scenario for Ethanol/Sulfate

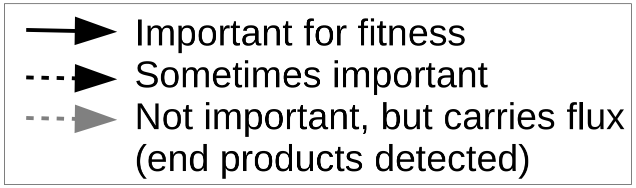

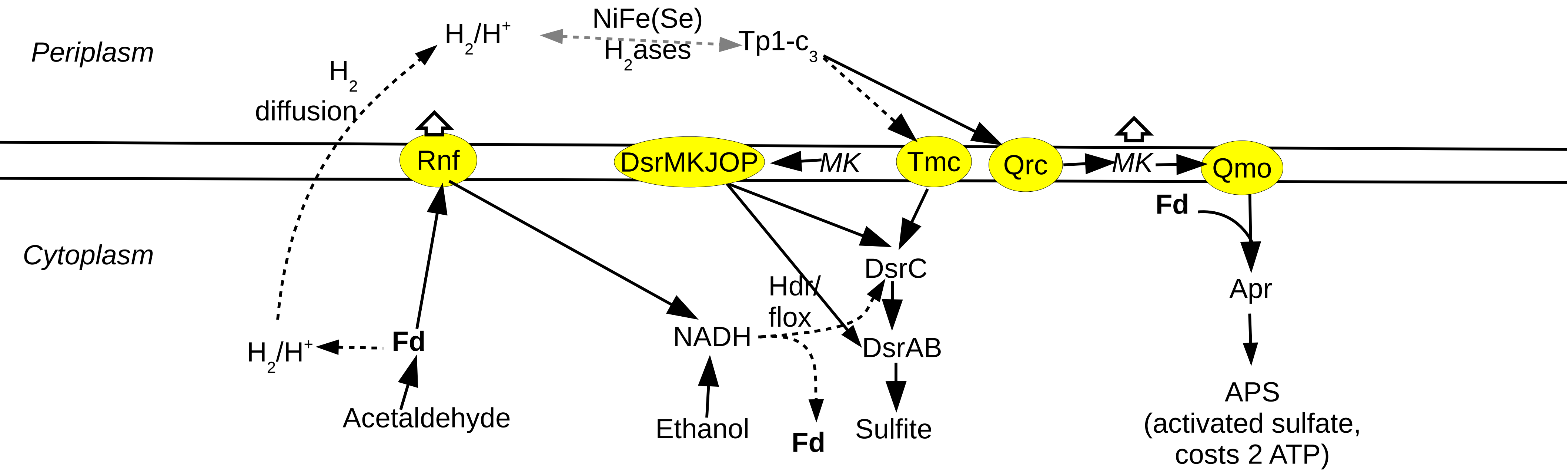

Per sulfate reduced:

- 2 ethanol → 2 acetaldehyde + 2 reduced NADH
- 2 acetaldehyde → 2 acetate + 2 reduced Fd
- No substrate-level phosphorylation

To reduce 1 APS, need 2.5 reduced MK, 1 reduced DsrC, 0.5 reduced Fd

- So need to convert 2 NADH + 1.5 Fd → 1 DsrC + 2.5 MK

Rnf together with Hdr/flox convert reduced Fd or NADH to reduced DsrC, with ion pumping

Qrc produces reduced MK, but how do electrons reach Tp1-c_3?

- H_2 cycling?
- DsrMKJOP could be an alternate source of reduced MK?
- Hmc? (but Hmc is detrimental to fitness)

Or, most of the electrons for sulfite reduction come from DsrC and not from MK?

Note: electron flow/sulfate on choline is expected to be similar, as choline is disproportionated to ethanol + acetyl-phosphate, but this allows for substrate-level phosphorylation (0 net ATP per sulfate reduced)

Scenario for H_2/Sulfate

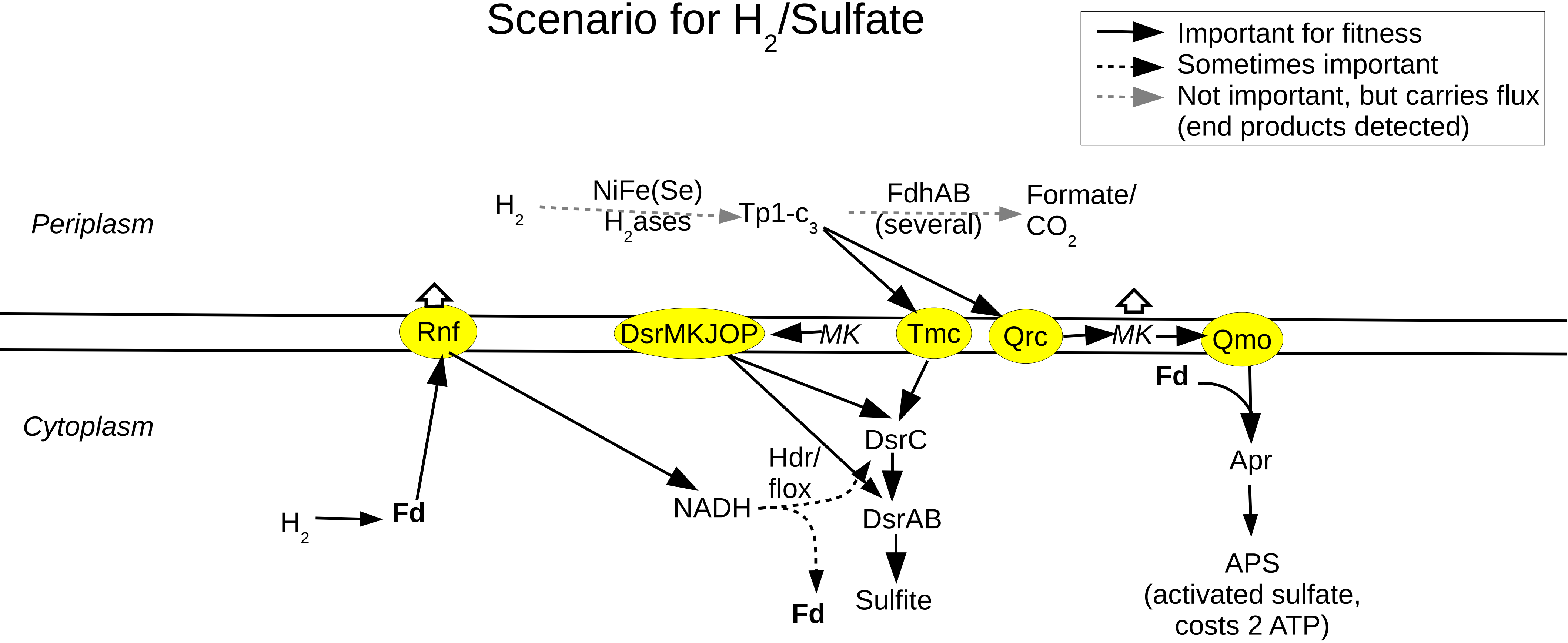

Per sulfate reduced:

- $4\ H2 \rightarrow 8\ H^+ + 4$ reduced Tp1-c_3, and/or
- $4\ H2 \rightarrow 8\ H^+ + 4$ reduced Fd
- No substrate-level phosphorylation

To reduce 1 APS need: 2.5 reduced MK, 1 reduced DsrC, 0.5 reduced Fd
Rnf together with Hdr/flox convert reduced Fd to reduced DsrC, with ion pumping
Qrc reduces MK (from Tp1-c_3)
Tmc reduces DsrC from Tp1-c_3)

Energy conservation by Rnf, Hdr/flox, and Qrc/Qmo

Scenario for Formate/Sulfate

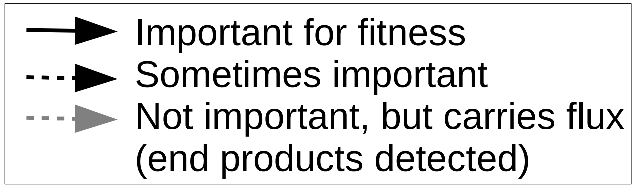

Overview:

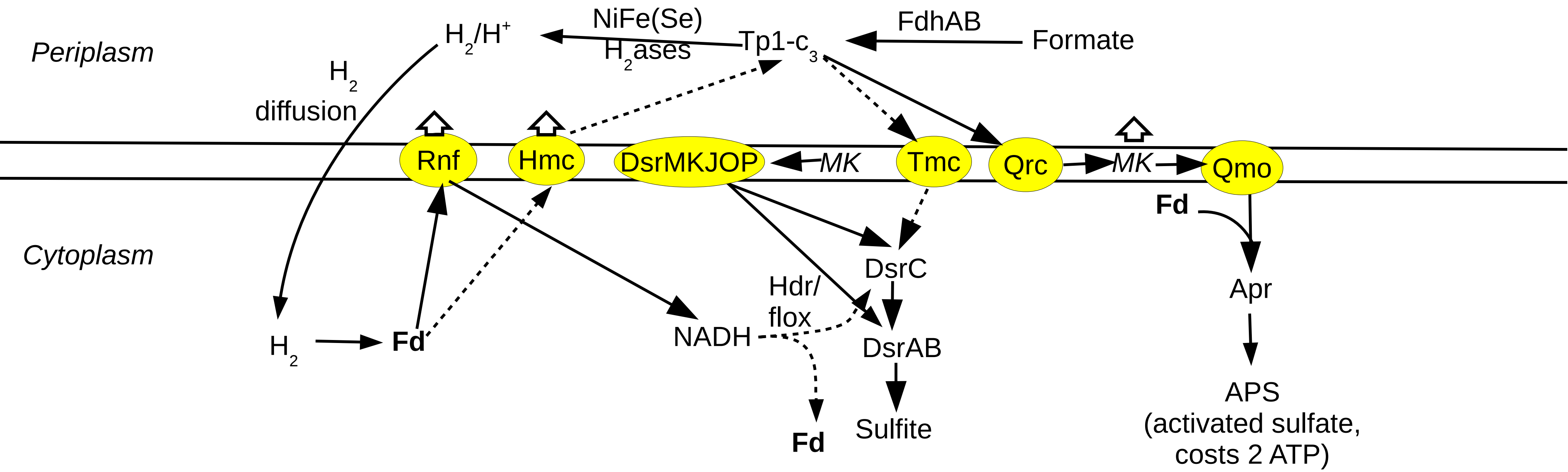

Per sulfate reduced:

    4 formate → 4 CO_2 + 4 reduced Tp1c_3

    No substrate-level phosphorylation

To reduce 1 APS, need 2.5 reduced MK, 1 reduced DsrC, 0.5 reduced Fd

Hmc can reduce Tp1-c_3 if oxidation is primarily cytoplasmic (due to cross-feeding of H_2)?

Qrc reduces MK

Conversion to H_2 and diffusion into cytoplasm allows reduction of Fd by cytoplasmic H_2ase

Rnf together with Hdr/flox can convert reduced Fd to reduced DsrC, with ion pumping

Or, Tmc produces reduced DsrC

Energy conservation by Rnf, Hdr/flox, Qrc/Qmo, and possibly Hmc
