# Supplementary material for: The genetic basis of energy conservation in the sulfate-reducing bacterium Desulfovibrio alaskensis G20
Source: Front Microbiol. 2014 Oct 31;5:577. doi: 10.3389/fmicb.2014.00577 (PMC4215793; doi:10.3389/fmicb.2014.00577)
Supplement: Supplementary file 2 [file Presentation2.PDF]

Supplementary Material for

The genetic basis of energy conservation in the sulfate-reducing bacterium Desulfovibrio alaskensis G20

Morgan N. Price[1], Jayashree Ray[1], Kelly M. Wetmore[1], Jennifer V. Kuehl[1], Stefan Bauer[2], Adam M. Deutschbauer[1], Adam P. Arkin[1,2,3]

1. Physical Biosciences Division, Lawrence Berkeley Lab, Berkeley, CA, USA.
2. Energy Biosciences Institute, University of California, Berkeley, CA, USA.
3. Department of Bioengineering, University of California, Berkeley, CA, USA.

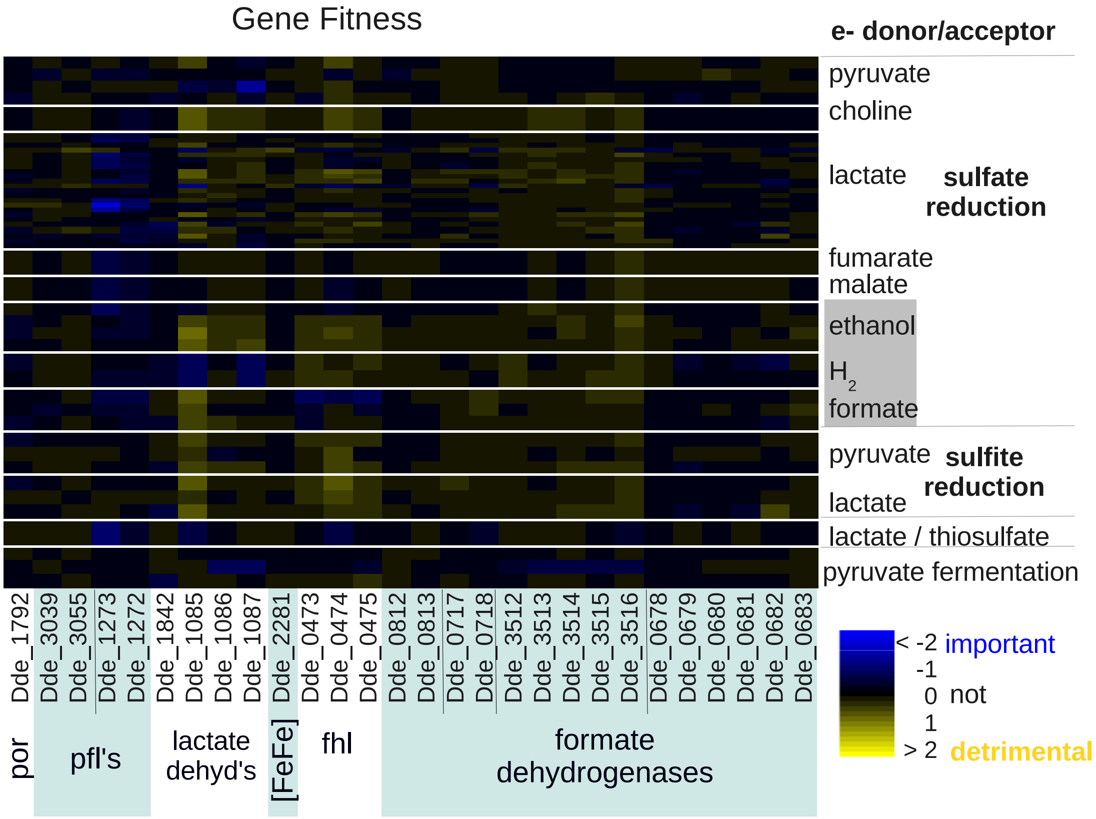

Figure S1. Heatmap of fitness data for genes that might relate to the utilization of various electron donors, but which lack strong phenotypes across 12 energetic conditions. por – pyruvate:ferredoxin oxidoreductase. pfl – pyruvate:formate lyase. [FeFe] – periplasmic iron-only hydrogenase. fhl – formate:hydrogen lyase.

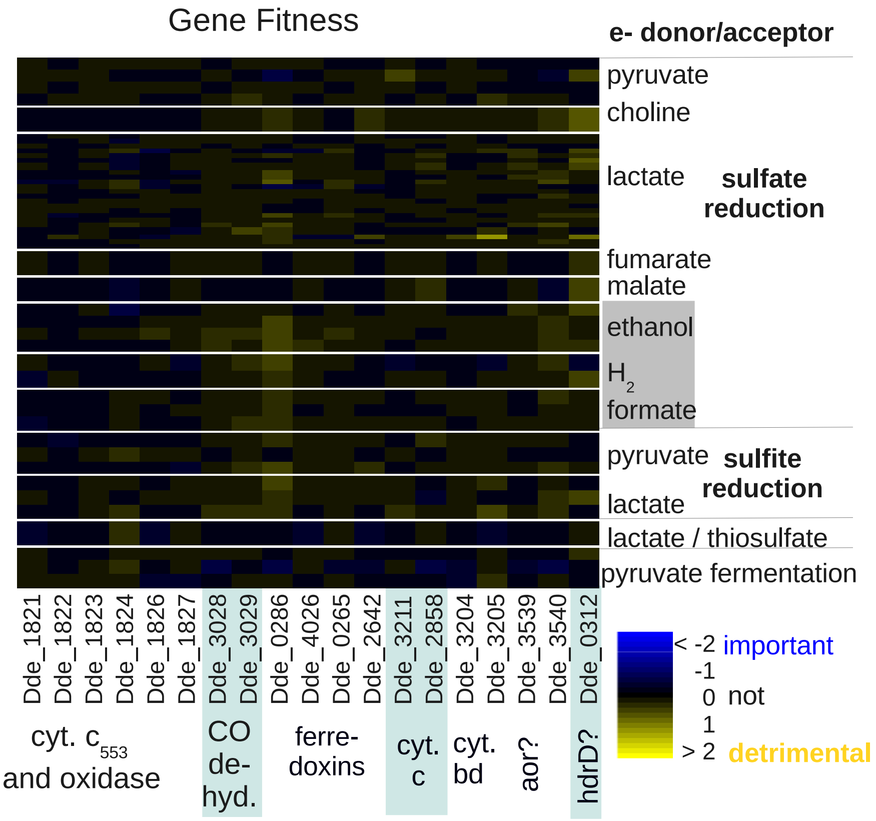

Figure S2. Heatmap of fitness data for electron transport genes that are not important for fitness in any of 12 energetic conditions.

Supplemental Data

Data Sheet 1: Metadata for the fitness experiments. Energy-related experiments are given in the same order as in the heatmaps, followed by additional experiments. Tab-delimited file:
http://morgannprice.org/G20energy/G20_energy_fitness_experiments.xls

Data Sheet 2: Per-strain fitness values. Tab-delimited file:
http://morgannprice.org/G20energy/G20_energy_strain_fitness.xls

Data Sheet 3: Per-gene fitness values. Tab-delimited file:
http://morgannprice.org/G20energy/G20_energy_gene_fitness.xls

Data Sheet 4: Mutants for follow-up studies and primers for confirming

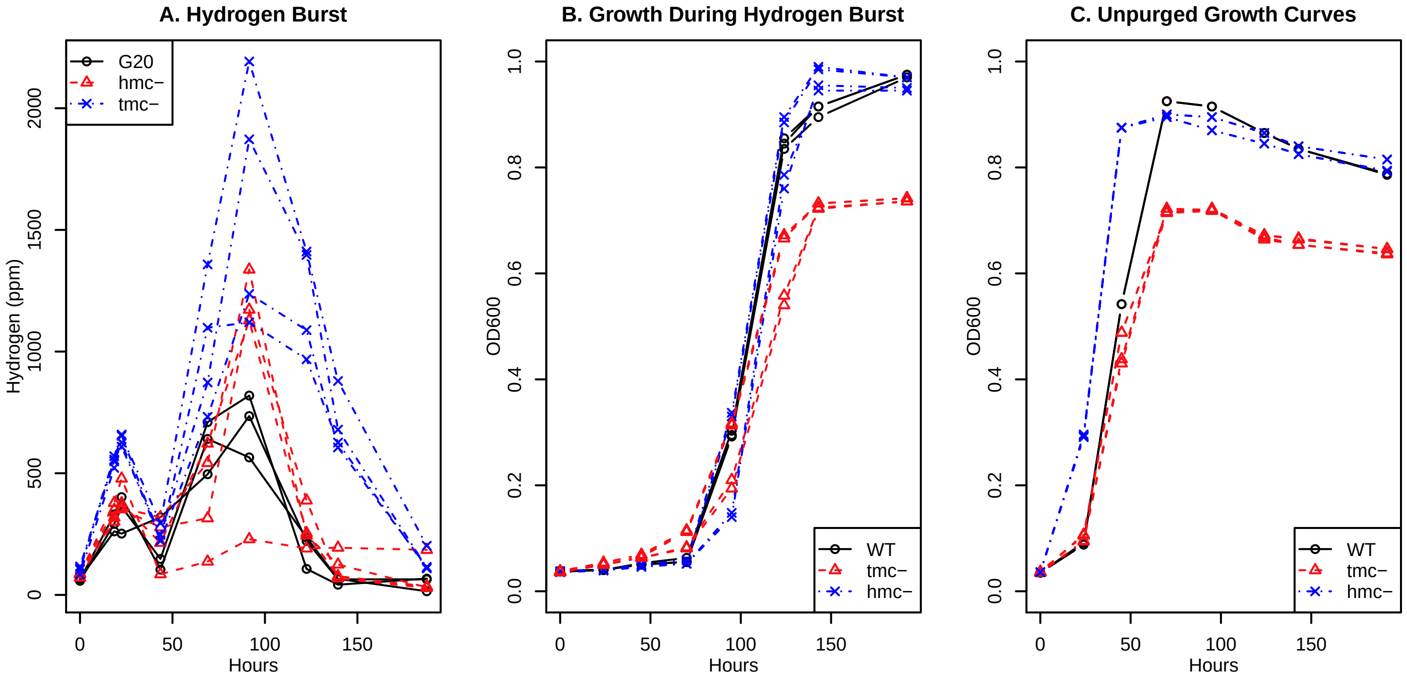

Figure S3: **(A)** Hydrogen levels in G20, *hmc* mutant, and *tmc* mutant strains growing in defined media with 60 mM lactate and 30 mM sulfate. The head space was purged with 80:20 N_2:CO_2 at the start of the experiment. The *hmc* mutants have insertions in *hmcE* or *hmcF*; the *tmc* mutants have insertions in *tmcB* or *tmcC*; and there are two replicates for each strain. **(B)** Growth during the hydrogen experiment. **(C)** Growth curves performed on the same day but without purging the headspace. Again, there are two replicates for each strain.

the transposon insertion location in each strain. Tab-delimited file: http://morgannprice.org/G20energy/Single_mutants.tab

Data Sheet 5: Concentrations (mM) of metabolites in the supernatant during growth of *D. alaskensis* G20 in defined media. Tab-delimited file: http://morgannprice.org/G20energy/G20_supernatant_concentrations.xls
